# Controlled Release of Agrochemicals Intercalated into Montmorillonite Interlayer Space

**DOI:** 10.1155/2014/656287

**Published:** 2014-02-17

**Authors:** Harrison Wanyika

**Affiliations:** Department of Chemistry, Jomo Kenyatta University of Agriculture & Technology, P.O. Box 62 000, Nairobi 00200, Kenya

## Abstract

Periodic application of agrochemicals has led to high cost of production and serious environmental pollution. In this study, the ability of montmorillonite (MMT) clay to act as a controlled release carrier for model agrochemical molecules has been investigated. Urea was loaded into MMT by a simple immersion technique while loading of metalaxyl was achieved by a rotary evaporation method. The successful incorporation of the agrochemicals into the interlayer space of MMT was confirmed by several techniques, such as, significant expansion of the interlayer space, reduction of Barrett-Joyner-Halenda (BJH) pore volumes and Brunauer-Emmett-Teller (BET) surface areas, and appearance of urea and metalaxyl characteristic bands on the Fourier-transform infrared spectra of the urea loaded montmorillonite (UMMT) and metalaxyl loaded montmorillonite (RMMT) complexes. Controlled release of the trapped molecules from the matrix was done in water and in the soil. The results reveal slow and sustained release behaviour for UMMT for a period of 10 days in soil. For a period of 30 days, MMT delayed the release of metalaxyl in soil by more than 6 times. It is evident that MMT could be used to improve the efficiency of urea and metalaxyl delivery in the soil.

## 1. Introduction

Agrochemicals are important ingredients for achieving global food security. However, the need for periodic application stemming from the huge losses witnessed due to volatilization, photodegradation, leaching, and surface migration may render agrochemical usage economically unsustainable [1]. Moreover, heavy usage of the agriculture chemicals has led to serious ecological pollution [2].

Agrochemical formulations which combine minimum amount of active ingredients (ai) with prolonged efficacy would effectively reduce postapplication losses. Important ways to achieve this are by photostabilisation and slowing the release of the active materials [3, 4].

Clay minerals are natural and relatively cheap components of soils. They are suitable materials for controlled release (CR) and photostabilized agrochemicals due to their high adsorption power, easily modified surfaces, and their colloidal nature [5]. Clay-based formulations of agrochemicals can reduce their leaching, photodegradation, and volatilization. Consequently, they can improve the efficiency of the agricultural chemicals and reduce environmental pollution [6]. Research by Gamiz et al. [7] shows organoclays as good amendment of soils to enhance its herbicide retention capacity.

Montmorillonite [(Na,Ca)_0.33_(Al,Mg)_2_(Si_4_O_10_)(OH)_2_·nH_2_O)] is a soft 2 : 1 layered phyllosilicate clay comprised of highly anisotropic platelets separated by thin layers of water. The platelets have an average thickness of ~1 nm and average lateral dimensions ranging between hundreds of nm and several *μ*m. Each platelet contains a layer of aluminium or magnesium hydroxide octahedral sandwiched between two layers of silicon oxide tetrahedral. The faces of each platelet have a net negative charge, which causes the interstitial water layer (known as the gallery) to attract cations (Ca^2+^, Mg^2+^, Na^+^, etc.). Much focus has been paid to MMT because of their strong adsorption capacities due to their high cation exchange capacity, (CEC), swelling capacity and high surface areas [8].

MMT have been used as a drug delivery system (DDS) previously. Recent research indicates that, with proper control of intercalation conditions, MMT can carry nicotine, timolol, ibuprofen, and donepezil among other therapeutic drugs and effectively modulate the drug release behavior. The drug releasing behaviour of MMT can also be modified by intercalation with other nanoparticle materials to form composites such as use of polymers including hydrogels, soluble polymers, and biodegradable and nonbiodegradable polymers [9].

*In vitro* studies by Cornejo et al. [10] indicated that MMT-based formulations of simazine released the herbicide slowly into aqueous solution. Celis et al. [11] reported high adsorption capacities for herbicide hexazinone by MMT saturated with Fe^3+^ and hexadecyltrimethylammonium cations.

Intercalation of agrochemicals into montmorillonite may offer multiple benefits. The gallery space would provide room for agrochemical molecules to occupy. The inorganic sheets would offer protection against environmentally induced rapid disintegration while intermolecular forces would ensure sustained release, consequently leading to long treatment periods for the same quantity of active ingredient [12].

Urea [carbonyl diamine] and metalaxyl [methyl N-(2,6-dimethylphenyl)-N-(2-methoxyacetyl) alaninate] were used as model fertilizer and pesticide molecules, respectively, to study the ability of MMT to store and controllably release agrochemical molecules. The choice of urea which constitutes about 40% of the total global nitrogen fertilizers application was made based on its high aqueous solubility, rapid hydrolysis by soil urease, and photo lability which has led to its considerable and rapid loss in soil [13]. Metalaxyl is an acylanilide fungicide with residual and systemic activity against fungi of the order Peronosporales, which attack a wide range of crops. Its application may be foliar, soil incorporation, surface spraying (broadcast or band), drenching, sprinkler or drip irrigation, or soil mix or seed treatment [14]. For the metalaxyl incorporated in soils to offer protection to crops against diseases for extended periods of time, migration rate of the fungicide due to leaching, photodecomposition and enzymatic degradation must be slowed down. Intercalation of metalaxyl into montmorillonite may offer a feasible approach to meet the aforementioned end.

In this work, commercial MMT was purified using sedimentation and centrifugation techniques. Urea and metalaxyl molecules were successfully incorporated into the interstitial space of MMT by employing simple emersion and rotary evaporation techniques. The clay/agrochemical composites were characterized by electron microscopy (SEM and TEM), Fourier-transform infrared (FT-IR) spectroscopy, powder wide angle X-ray diffraction (WAXRD), N_2_ sorption studies, and thermal gravimetric analysis/differential thermal analysis (TGA/DTA). Controlled release properties of the loaded complexes were studied in water and in the soil.

## 2. Materials and Methods

All the chemicals used in this study were of high purity. Montmorillonite K10 was purchased from Alfa Aesar, USA; metalaxyl Pestanal (99.9%), P-dimethylaminobenzaldehyde, urea (>99%), hydrochloric acid, and trichloroacetic acid (99.0%) were purchased from Sigma-Aldrich, UK. All the reagents apart from montmorillonite were used as purchased without further purification. Water used in the experiments was purified by Millipore Milli-Q system to a resistivity of 18.2 MΩ·cm.

### 2.1. Isolation of Montmorillonite Nanoparticles

Montmorillonite nanoparticles (MMT NP) were obtained from commercial MMT. Particles having diameter greater than 2 *μ*m were removed by sedimentation for 12 hours. The smaller MMT particles were removed by suction from the coarse settled minerals. Sedimentation was repeated two times. Nanoparticles were isolated by centrifugation for 15 minutes at 10000 rpm. They were then oven-dried at 120°C for 3 hours.

### 2.2. Intercalation of Urea into MMT

Preliminary tests were conducted on the as-purchased MMT denoted by MMT MP and MMT NP using varied concentrations of urea. MMT (20 mg) samples were suspended in 4 mL of 0.17, 1.67, and 8.33 moles/litre aqueous urea solutions for 24 hours at RT using a magnetic stirrer at 300 rpm. The suspensions were centrifuged at 10 000 rpm for 10 min and urea concentration of the supernatant was determined by UV/Vis spectrophotometry method described by Wanyika et al. [13]. The difference between initial concentration and final (supernatant) concentration was construed to be the sum of urea intercalated into the interlayer and urea adsorbed on the MMT surface.

Based on initial preliminary results, MMT NP and 8.33 moles/litre aqueous urea solutions were chosen for all the subsequent experiments. First, equilibration time was determined by suspending 20 mg of MMT NP into 4 mL of urea solution for 15 min, 30 min, 60 min, 100 min, 200 min, and 300 minutes, followed by determination of the urea content in the supernatant. Secondly, using an excess of established equilibration time, an experiment to study the reaction kinetics was conducted with 20 mg of MMT NP and 4 mL of urea solution for 1 min, 5 min, 10 min, 15 min, 20 min, 30 min, 45 min and 60 minutes. Finally, MMT NP (500 mg) was dispersed in 20 mL of 8.33 moles/litre aqueous urea solutions for 24 hours at room temperature using a magnetic stirrer at 300 rpm. The suspensions were centrifuged at 10 000 rpm for 10 min and then oven-dried at 80°C for 4 hours. The urea-MMT NP composite denoted by UMMT was used for characterization and release studies.

### 2.3. Intercalation of Metalaxyl into Montmorillonite Interlayer Space

MMT NP (230 mg) was suspended in 20 mL of water containing 9.8 mg of metalaxyl. Dissolution of metalaxyl in water was facilitated by sonication for 10 minutes. The suspension was stirred for 2 hours, and then the solvent was evaporated to ~5 mL using a rotary evaporator at 45°C and reduced pressure. The MMT NP intercalate was isolated by centrifugation and the product, denoted by RMMT, dried at RT for 24 hours.

### 2.4. Characterization

Electron microscope images were collected on scanning electron microscope (SEM, Hitachi S4800) and transmission electron microscope (TEM, Tecnai G^2^ 20 S-TWIN). Infrared (IR) spectra were recorded on a FT-IR spectrophotometer (SPECTMM ONE B) using KBr discs in the range of 400–4000 cm^−1^. X-ray powder diffraction patterns (XRD) were obtained on a diffractometer (LR 39487C XRD) using Ni-filtered Cu K*α* radiation at 50 kV and 300 mA at a scanning speed of 4° min^−1^. Thermal analyses, that is, thermal gravimetric analysis (TGA) and differential thermal analysis (DTA), were carried out at a heating rate of 3° min^−1^ with Al_2_O_3_ using thermal gravimetric analyzer (TGA-7) from RT to 800°C. The nitrogen adsorption/desorption isotherms were collected on Micromeritics equipment (ASAP 2020). Specific surface areas and pore volumes were determined by standard BET and BJH methods respectively, from adsorption branches of the isotherms.

### 2.5. Controlled Release Experiments

#### 2.5.1. Controlled Release Behaviour of UMMT in Water

The static release profiles were studied for a period of 10 days with samples collected and analyzed daily. All the 10 measurements were started simultaneously. UMMT (10.0 mg) was accurately weighed. It was suspended in 3 mL of pure water in conical bottles. The suspensions were stirred at room temperature and centrifuged after every time lapse. Urea content of the supernatant was determined using the UV-Vis method previously described.

#### 2.5.2. Controlled Release Behaviour of UMMT in Soil

UMMT (26 mg) and a physical mixture comprising 11.7 mg urea and 14.3 mg MMT were well mixed with 18 g of dry soil (<2 mm in diameter) and put in 25 mL separating funnels at room temperature and water (12 mL) was added. Control experiment was done with 11.7 mg of pure urea mixed with 18 g of the soil while blank experiment was done with 18 g of soil alone. A fraction of water (5 mL) was eluted after every 24 hours; 5 mL of pure water was added back in order to maintain a constant volume of water slightly above the soil surface throughout the experiment. The elute was filtered through 0.22 *μ*m syringe filters and centrifuged, and urea content was estimated by UV/Vis method.

#### 2.5.3. Batch Release Kinetics of RMMT in Water

The release of metalaxyl from RMMT was carried out by suspension of 8.0 mg of RMMT in 4.0 mL of water contained in glass bottles and sealed with screw caps for different time periods. Different experiments for the different periods of time were set up simultaneously. In all cases, the release kinetics was obtained in triplicate. After each time lapse, the bottles were hand-shaken and centrifuged at 10 000 rpm for 10 minutes. The supernatant was filtered and analyzed by high performance liquid chromatography (HPLC). The following HPLC conditions were used: detector, UV, *λ* = 220 nm; column, supelcosil LC-18 5 *μ*m, 250 mm × 4.6 mm; temperature, 30°C; mobile phase, acetonitrile/water (50/50 v/v); injection volume, 10 *μ*L; flow rate, 1.0 mL min^−1^; run time, 10 minutes.

#### 2.5.4. Release of Metalaxyl in Soil

The controlled release characteristics of the RMMT were determined with soil filled glass separating funnels with a volume of 25 cm^3^. Pure metalaxyl (1.6 mg) corresponding to the amount entrapped in the RMMT was used as control. The soil columns were settled by the addition of 20 mL of water, 50 mg of RMMT was placed on top of soil columns, and 10 mL of pure water was applied on a three-day interval for one month to the tubes. The elutes were filtered through 0.22 *μ*m syringe filters and analyzed for metalaxyl content by HPLC method previously described.

## 3. Results and Discussion 

### 3.1. Purification of MMT

**Figure 1:**
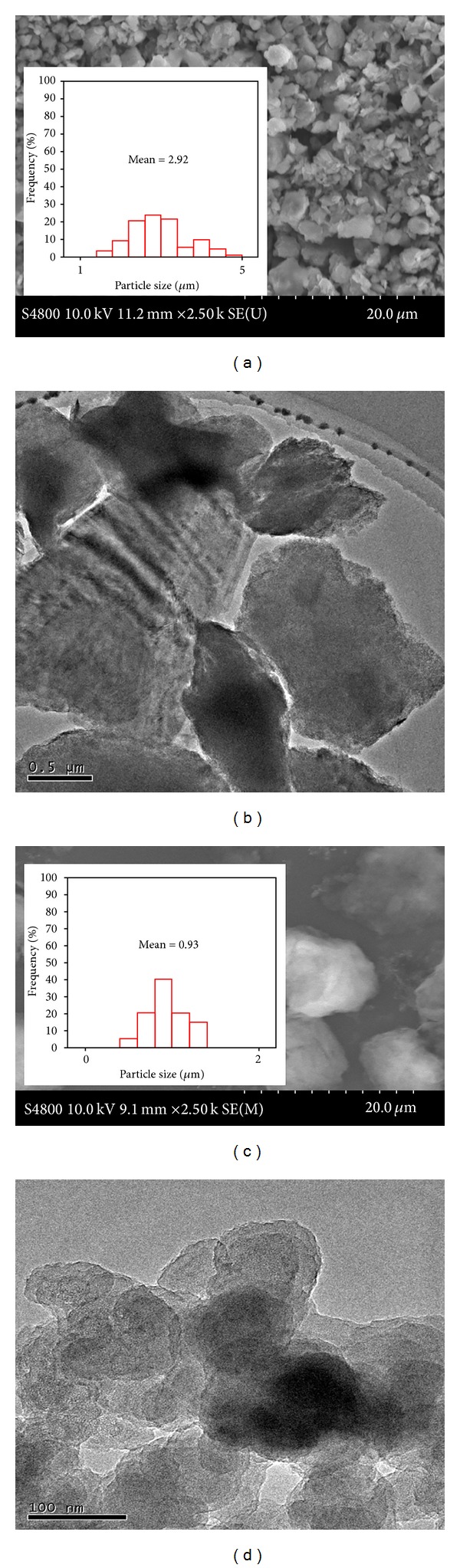
SEM and corresponding TEM images of (a)-(b) as-purchased MMT and (c)-(d) MMT NP. Inset: particle size distributions.

Electron microscopy images and corresponding particle size distributions of as-purchased MMT (MMT MP) and purified MMT (MMT NP) are displayed in Figure 1. As-purchased MMT consisted of wide particle size distribution averaged at 2.92 *μ*m. The purified MMT particle sizes ranged from ~550 nm to 1.3 *μ*m and averaged at 900 nm. It is clear from the microscopy observations that the purification process eliminated big particles and narrowed the particle size distributions.

### 3.2. Efficiency of MMT MP and MMT NP to Adsorb Guest Molecules

**Figure 2:**
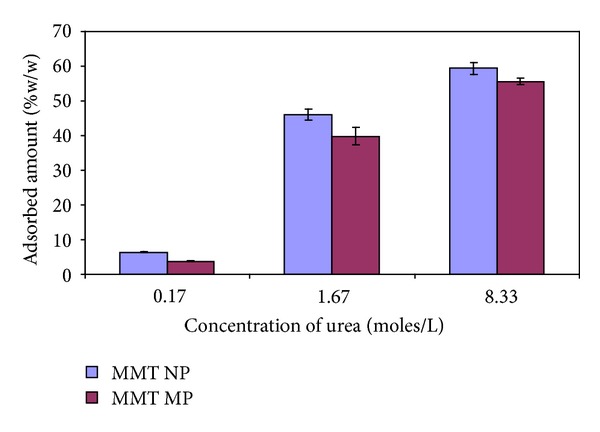
Efficiency of MMT NP and MMT MP in adsorption of urea.

Results on the preliminary adsorption of urea onto MMT MP and MMT NP are shown in Figure 2. MMT MP adsorbed 3.8 ± 0.4, 39.9 ± 10.5, and 55.6 ± 4.3 wt% urea from urea solutions with concentrations of 0.17, 1.67, and 8.33 moles/litre, respectively, while MMT NP adsorbed 6.4 ± 0.4, 46.0 ± 7.0, and 59.4 ± 7.5 wt%, respectively, from the same urea concentrations. The amount of urea adsorbed by both MMT samples increased significantly with increase in the concentration of the urea solution due to increased driving concentration gradient. MMT NP adsorbed more urea than the MMT MP due to high surface to volume ratio thus providing more room for the urea molecules to occupy. Furthermore, short diffusion paths enable smaller particles to be loaded easily and fast. All the subsequent experiments with urea and even metalaxyl were done with MMT NP due to the inherent advantages of dealing with small particles with narrow particle size distribution.

### 3.3. Urea Adsorption onto MMT Kinetics

**Figure 3:**
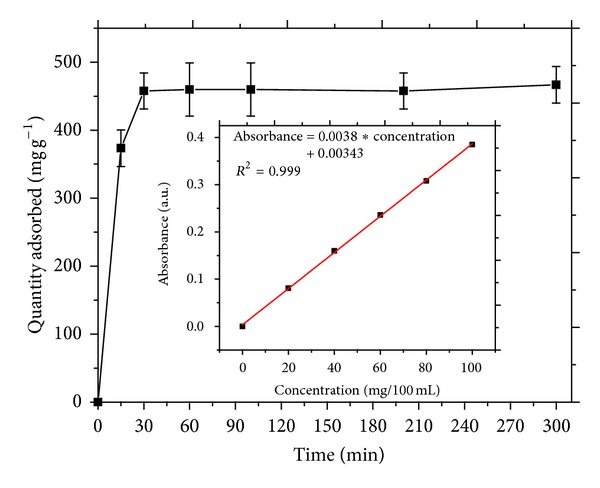
Time dependence of urea adsorption by MMT NP. Inset: calibration curve for urea determination.

**Figure 4:**
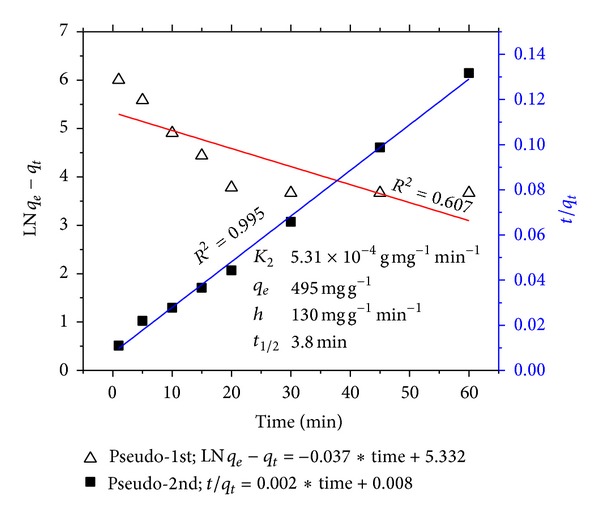
Pseudo-1st and -2nd order sorption kinetic models for intercalation of urea molecules into MMT NP interlayer. Inset: pseudo-2nd order kinetic parameters.

The time dependence of urea adsorption by MMT NP is exhibited in Figure 3. Equilibration time was found to be achieved within 30 minutes. The pseudo-1st and -2nd order kinetics models are shown in Figure 4. Adsorption of urea was found to follow pseudo-2nd order kinetics with an R^2^ value of 0.995 which implied that multiple adsorption sites were available for the guest molecules. The amount of urea adsorbed at equilibrium concentration was 495 mgg^−1^. This value indicated ~50 wt% adsorption efficiency. However, most of the urea was adsorbed on the outer surfaces of the MMT NP with fewer amounts being intercalated into the interlayer space.

### 3.4. Intercalation of Urea and Metalaxyl into MMT NP Interlayer

**Figure 5:**
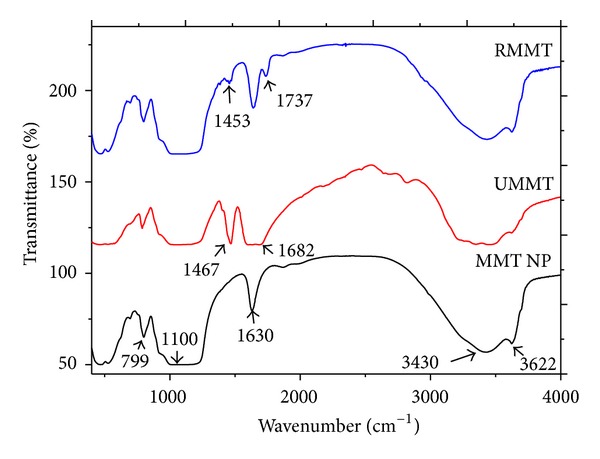
FT-IR spectra of MMT NP, UMMT, and RMMT.

FT-IR spectra of purified MMT, UMMT, and RMMT hybrids are shown in Figure 5. MMT NP spectrum exhibits major vibrational bands for pure MMT at 3622 cm^−1^ attributed to –OH stretching mode for Al–OH, 3430 cm^−1^ due to –OH stretching mode for interlayer water, 1630 cm^−1^ attributed to OH bending vibration of adsorbed water, and 1100 cm^−1^ corresponding to Si–O stretching vibration. UMMT spectrum reveals urea characteristic stretching frequencies of N–H and C=O at 1467 cm^−1^ and 1682 cm^−1^ which suggested presence of urea molecules within the MMT surface. Despite the fact that many metalaxyl characteristic modes were obscured by the strong MMT bands, two peaks at 1737 cm^−1^ attributed to the metalaxyl ester carbonyl stretching vibration and 1453 cm^−1^ assigned to disubstituted amide are clearly notable. Their presence indicates that metalaxyl was successfully adsorbed on MMT. Absence of foreign peaks from the UMMT and RMMT spectra suggested adsorption was by physical forces. Neutral organic molecules (soluble in water) are intercalated into the MMT interlayer by being trapped into the inorganic layers through Van der Waals interactions between the organic molecules and the siloxane surface [15].

### 3.5. Confirmation of Successful Intercalation

**Figure 6:**
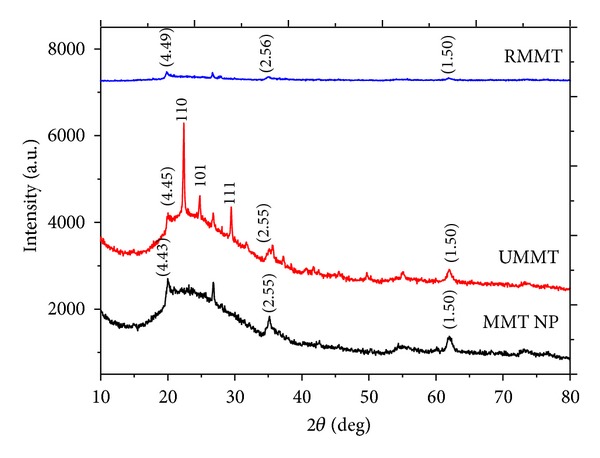
WAXRD patterns of MMT NP, UMMT, and RMMT.

WAXRD patterns of MMT NP, UMMT, and RMMT are given in Figure 6. Comparison of the experimental patterns with spectra from the spectral library enabled the identification of three MMT characteristic peaks which occurred at ~20°, 35^°,^ and 62° 2*θ* (Figure 6). Intercalation of urea into MMT interlayer space was confirmed by the increase of the basal spacing of the main X-ray diffraction peak (~20° 2*θ*) from 4.43 to 4.45 Å. Some urea that was not entrapped into the pores crystallized into separate crystals on the surface of MMT as revealed by presence of (110), (101), and (111) Bragg reflections for urea's tetragonal crystal system. The intensity of the MMT NP peaks was diminished after loading with metalaxyl. Furthermore, the basal (d) spacings of the major peak at ~20° 2*θ* increased from 4.45 Å to 4.49 Å. Intercalation of the agrochemical molecules could also have been facilitated by complexation with metals in the interlamellar of the MMT [12]. This XRD finding propounded a rearrangement in the interlayer space due to the presence of the fungicide which was verified by nitrogen sorption studies.

### 3.6. Effect of Intercalation on the Physicochemical Parameters of MMT

**Figure 7:**
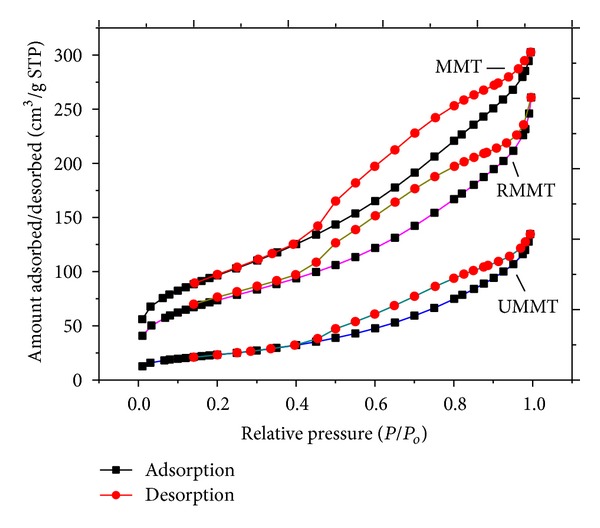
N_2_ adsorption-desorption isotherms of MMT, UMMT, and RMMT.

**Figure 8:**
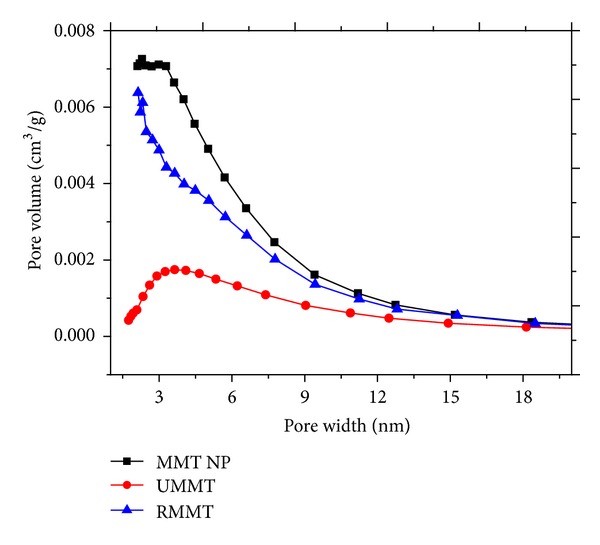
BJH pore size distributions of MMT NP, UMMT, and RMMT.

Nitrogen sorption isotherms and their corresponding pore size distributions are displayed in Figures 7 and 8, respectively. A summary of the derived physicochemical properties is presented in Table 1. After interaction between the purified MMT, urea, and metalaxyl molecules in solution, the BET surface area (*S*_BET_) and BJH total pore volume (*V*_*T*_) of MMT NP decreased from 311 m^2^/g and 0.5 cm^3^/g, respectively, to 81 m^2^/g and 0.21 cm^3^ for UMMT while RMMT decreased to 267 m^2^/g and 0.42 cm^3^/g. The average pore size increased from 5.7 nm to 7.9 nm for UMMT and to 6.5 nm for RMMT. The changes in physicochemical parameters indicated successful adsorption of the agrochemicals more important, the expansion of pore size confirmed further that intercalation of the agrochemical molecules into the interlamellar space had taken place. Indeed, the molecular diameter of metalaxyl molecule was estimated by materials studio software to be ~0.8 nm which correlated well with the magnitude of interlayer space expansion.

### 3.7. Amount of Agrochemicals Intercalated

**Figure 9:**
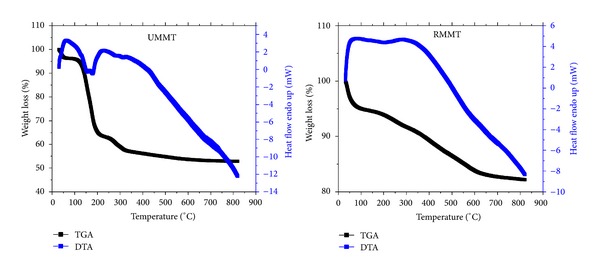
TGA/DTA curves of UMMT and RMMT.

**Figure 10:**
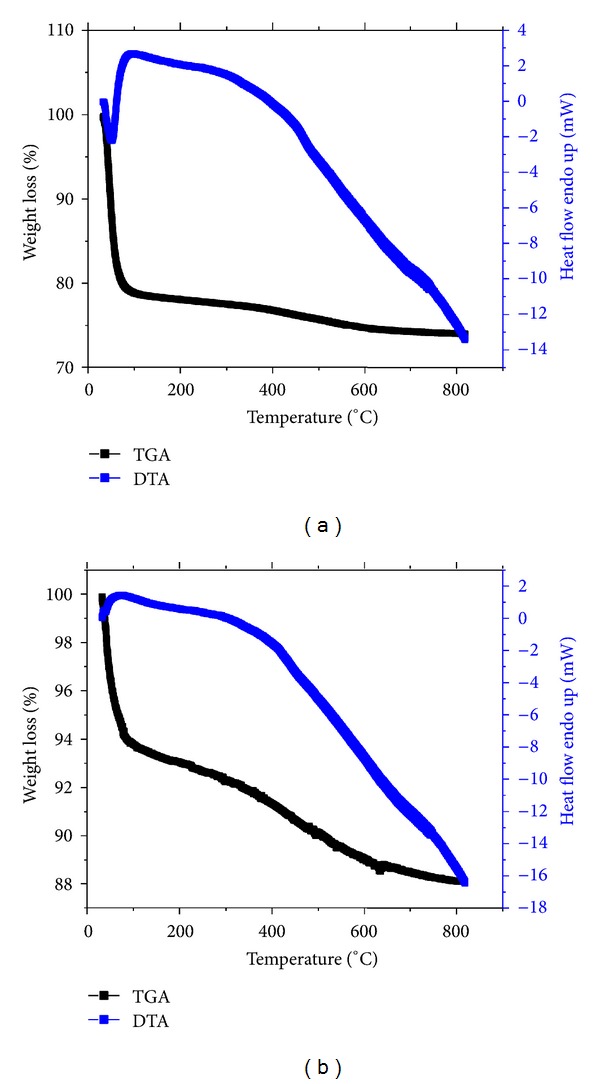
TGA/DTA curves for MMT NP used in (a) UMMT and (b) RMMT.

TGA/DTA thermograms of UMMT and RMMT are exhibited in Figure 9. Thermograms for corresponding MMT NP are shown in Figure 10. Unloaded MMT NP used for urea and metalaxyl lost 25.7% and 11.8%, respectively, of its weight ascribed to desorption of water adsorbed in the interlayer space while UMMT and RMMT lost 47.1% and 19.9%, respectively. Amount of urea and metalaxyl intercalated into MMT NP, calculated as the difference between the two was found to be 21.4 wt% and 8.1 wt%, respectively. The high amount of urea adsorbed in the interlayer region could be due to the hydrophilic nature of the urea molecules that enabled it to bond more strongly (by hydrogen bonds and Van der Waals interactions) with the charged clay surfaces. Conversely, metalaxyl is more hydrophobic and therefore its interaction with clay surface is largely by Van der Waals forces.

### 3.8. Sustained Release of UMMT in Water and Soil

**Figure 11:**
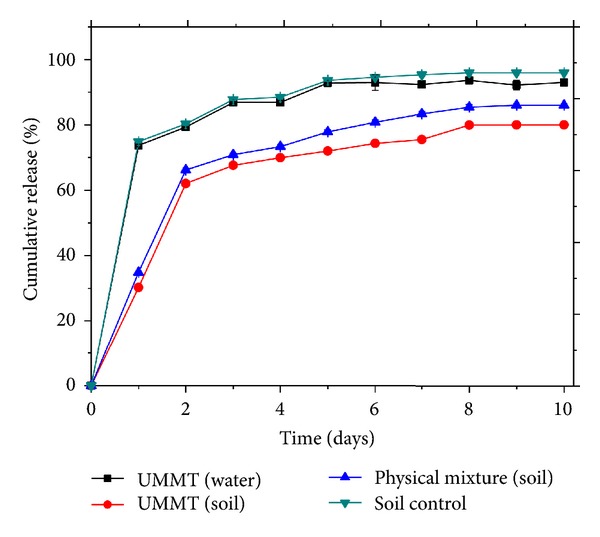
Time dependence release of urea from UMMT nanocomposite in water and soil.

**Figure 12:**
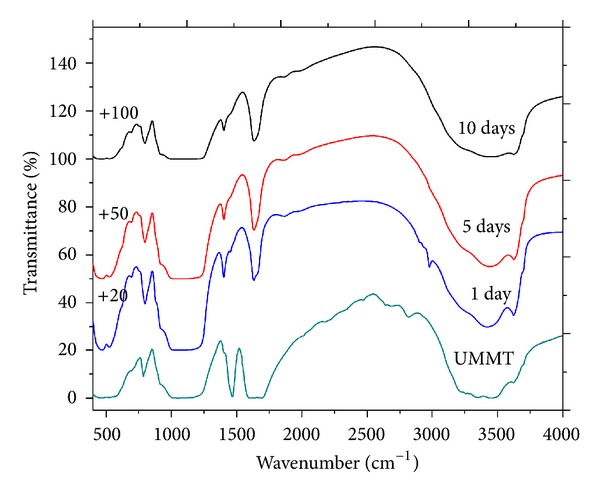
IR spectrum of UMMT and corresponding spectra after releasing urea in water for different days.

The SR profile of UMMT in water and in soil is exhibited in Figure 11.  Figure 12 reveals residual urea in the MMT NP matrix after suspending UMMT in water for the entire study period. Though UMMT did not release all the urea at once in water, the rate of release was found to be rather fast with ~74% w/w released after day 1 and ~93% w/w after day 10. This was as a result of the high solubility of urea in water that made it possible for urea molecules to be quickly displaced by water molecules from the gallery space. Presence of residual urea molecules after release indicated stronger interaction of some urea molecules with MMT NP. Free urea (control) was released fast with ~75% w/w released within one day and ~96% w/w by the end of 10 days in soil. For urea molecules intercalated into MMT NP, only ~30% w/w and 80% w/w were released in soil after 1 and 10 days, respectively. A physical mixture between urea and MMT NP that was used to assess whether mere adsorption on the outside surfaces of MMT NP was sufficient for delayed release performed significantly worse than UMMT but slightly better than free urea with ~35% released within day one and ~86% w/w after 10 days. Obviously, urea in the gallery space of MMT NP was solvated by water molecules in the soil into a complex which together with the effects of intermolecular forces of attraction contributed to the delayed release. Although the physical mixture of urea and metalaxyl did not have any possibility of intercalation, it performed better than free urea because of increased clay content in the soil which led to increased urea adsorption capacity thus reducing its rate of migration.

Urea molecules located within the interlayer space of MMT NP could be protected against decomposition by photochemical, thermal, enzymatic, and other catalytic activities of soils unlike free-urea molecules on the surface of soil particles. When they are in contact with soil water and adsorbed water of soil particles, they are readily transferred into the waters by diffusion. When they are hydrolyzed to ammonia, MMT can quickly adsorb urea-derived ammonia through physical and chemical interactions not only because MMT has both high water holding and cation exchange capacities but also because MMT and the ammonia tend to be located very closely. Therefore, MMT is expected to play an essential role in suppressing emission of ammonia through delivery of urea into inner soils and adsorption of ammonia.

### 3.9. Sustained Release of RMMT in Water and Soil

**Figure 13:**
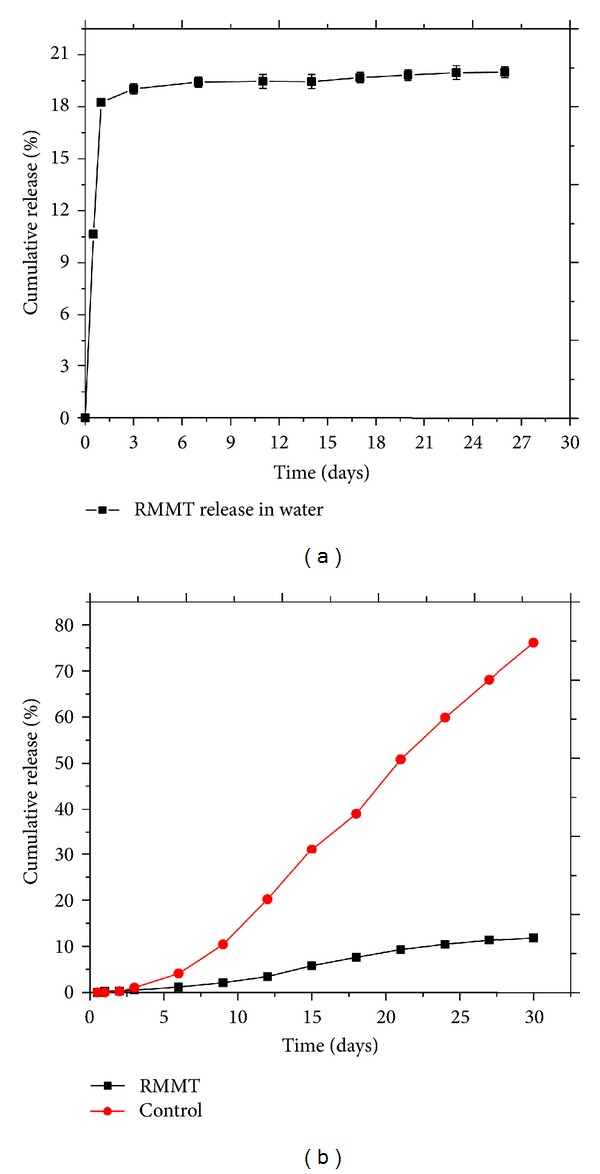
Release profiles of RMMT in (a) water and (b) soil.

The release profile of RMMT in water and in soil is shown in Figure 13. About 20% w/w of the intercalated metalaxyl was released in water within a period of 26 days. In soil experiment, only ~11.9% w/w was released within one month while control experiment in the soil registered ~76.1% w/w release within the same period. The slight delay in release of free metalaxyl in soil was due to its low solubility in water coupled with adsorption in the soil. Drastic improvement in release period was experienced with RMMT. The findings indicated that cumulative release of metalaxyl from MMT occurred in a controlled and sustained manner.

## 4. Conclusions 

Intercalation of urea and metalaxyl into purified MMT was achieved. The entrapped/intercalated molecules possess the same physical and chemical properties with those in free state. Sorption kinetic studies demonstrated that the carrier materials could adsorb high concentrations of the agrochemicals by physisorption. High adsorption efficiency would facilitate the use of less delivery carriers and prolong the release period. Physical adsorption ensures that the chemical nature of the guest molecules is not altered.

The release of the intercalated molecules was significantly retarded which is sine qua non for controlled release application.

## Figures and Tables

**Table 1:** Physicochemical properties of MMT NP, UMMT and RMMT.

	*S*_BET_ (m^2^/g)	*V*_*T*_ (cm^3^/g)	Pore size (nm)
MMT NP	311	0.50	5.7
UMMT	85	0.21	7.9
RMMT	267	0.42	6.5
